# Illness-related behaviour and utilization of oral health services among adult city-dwellers in Burkina Faso: evidence from a household survey

**DOI:** 10.1186/1472-6963-6-164

**Published:** 2006-12-27

**Authors:** Benoît Varenne, Poul Erik Petersen, Florence Fournet, Philippe Msellati, Jean Gary, Seydou Ouattara, Maud Harang, Gérard Salem

**Affiliations:** 1 UR178, Institut de Recherche pour le Développement, 01 BP 182 Ouagadougou 01, Burkina Faso; 2 World Health Organization Global Oral Health Programme, Department of Chronic Disease and Health Promotion, World Health Organization, 20, Avenue Appia, CH-1211 Geneva 27, Switzerland; 3 UMR145, Institut de Recherche pour le Développement, 01 BP 171 Bobo Dioulasso 01, Burkina Faso; 4 Centre Muraz, Ministry of Health of Burkina Faso, 01 BP 390 Bobo-Dioulasso 01, Burkina Faso; 5 Université Paris X Nanterre, Laboratoire Espace, Santé et Territoire, 200 Avenue de la République, 92001 Nanterre Cedex, France

## Abstract

**Background:**

In sub-Saharan Africa, the availability and accessibility of oral health services are seriously constrained and the provision of essential oral care is limited. Reports from the region show a very low utilization of oral health care services, and visits to dental-care facilities are mostly undertaken for symptomatic reasons. The objectives of the present study were to describe the prevalence of oral symptoms among adults in Ouagadougou, capital city of Burkina Faso and the use of oral health services and self-medication in response to these symptoms and to measure the associations between predisposing, enabling and needs factors and decisions to seek oral health care.

**Methods:**

The conceptual design of the study was derived from both the Andersen-Newman model of health care utilization and the conceptual framework of the WHO International Collaborative Study of Oral Health Outcomes. Data were obtained by two-stage stratified sampling through four areas representative of different stages of urbanization of Ouagadougou. The final study population comprised 3030 adults aged 15 years or over and the response rate was 65%.

**Results:**

Overall, 28% of the respondents had experienced an oral health problem during the past 12 months; a high proportion (62%) reported pain or acute discomfort affecting daily life. In response to symptoms, only 28% used oral health facilities, 48% used self-medication and 24% sought no treatment at all. Multivariate analyses revealed that several socio-economic and socio-cultural factors such as religious affiliation, material living conditions and participation in a social network were significantly associated with the use of oral health care services by adults who had experienced oral health problems during the previous year.

**Conclusion:**

The proportion of people who have obtained oral health care is alarmingly low in Ouagadougou and self-medication appears to be an important alternative source of care for adult city-dwellers. Decision-makers in sub-Saharan countries must seek to ensure that access to essential oral health care is improved.

## Background

In sub-Saharan Africa, communicable diseases are still major threats to health but the burden of chronic diseases and injuries are expected to increase rapidly [1]. The epidemiological transition in relation to oral health is particularly apparent in the growing incidence of dental diseases such as caries and periodontal conditions. Other conditions such as oral cancer, oral manifestations of HIV/AIDS and dental trauma are also expected to increase in the near future [2].

In many African countries, the availability and accessibility of oral health services are seriously constrained, and the provision of essential oral care is limited. Many studies have been undertaken in industrialised countries to assess the level and the pattern of utilization of dental health services [3,4]. Unfortunately, this has been a neglected field of research in Africa. The few existing reports from the region show very low utilization of oral health care services, and visits to a dental-care facility are mostly undertaken for symptomatic reasons [5-8]. A survey conducted in a peri-urban settlement of South Africa found that 37.0% of adults had consulted a dentist or medical practitioner, usually for tooth extractions [6]. A study in the Ivory Cost reported that only 11.4% of the city-dwellers in Abidjan had visited a dentist because of dental problems [7]. Another study in Nigeria showed that 9.0% of households had used dental services during the past year while variables such as "zone of residence" and "household educational and social class ranking" affected use of oral care services [8]. In Tanzania, the principal factors associated with the utilization of oral health care services were distance to treatment facility and previous dental symptoms [9]. Concomitantly, self-medication with herbal medicines or modern drugs seems to be a common practice in many African countries [10,11].

Systematic studies of the utilization of oral health services and the factors involved in seeking health care are urgently needed so that oral health programmes may be developed to match the needs of the population. The objectives of the present study were to describe the prevalence of oral symptoms among urban adults and the use of oral health services and self-medication in response to these symptoms, and to analyze the associations between predisposing, enabling and needs factors and decisions about seeking oral health care. This study is part of a larger research programme carried out by the Institut de Recherche pour le Développement (IRD) to assess social and spatial health disparities in Ouagadougou.

## Methods

### Study area

Ouagadougou, the capital city of Burkina Faso, is experiencing an urban population boom, with expected increases in poverty and health disparities typical of many other major towns in sub-Saharan Africa [12,13]. Between the years 1980 and 2005, the population of Ouagadougou grew more than four-fold, from 280,000 to 1,200,000 inhabitants. More than 25.0% of the population currently live in irregular peri-urban settlements of the town characterized by poorly constructed houses, poor sanitary conditions and lack of all services.

Like the general health services, oral health care in Burkina Faso has been organized through a public/private mixture of providers based on an out-of-pocket payment system. The national health insurance system does not cover any costs of curative dental care. The dentist to population ratio for the whole country is one per 220,000. Oral health facilities in the capital city are somewhat better equipped and definitely more numerous than in other parts of the country. In 2005, 24 oral health care services were established in Ouagadougou: 14 facilities within the private health sector, 4 within the public health sector and 6 within the nongovernmental not-for-profit health sector [14]. In parallel, a large number of pharmacies, street-market traders and traditional healers play a significant role in providing oral health care.

### Study design and sample

To tackle the complex health problems resulting from environmental and social changes in an urban setting, the whole research approach has to be multi-disciplinary in accordance with epidemiological and geographical constraints.

Because of heterogeneity of ecological situations within the capital city and the absence of recent socio-demographic census data, a two-stage stratified sampling technique was used. The purpose was to identify various ecological settings representative of the distinct levels of urbanization in Ouagadougou. The stratification process was based on two criteria: building density, by comparing "average building density" with "high building density"; and settlement subdivision, by comparing "regular settlements" with "irregular settlements". Building density, the first criterion of stratification, was evaluated using a SPOT 5 (Satellite Pour l'Observation de la Terre) panchromatic satellite image of Ouagadougou dated November 26, 2002. In brief, ERDAS Imagine^© ^software was used to locate buildings, and Arc GIS 8.2^© ^made it possible to calculate the density of the built-up area. The process was supplemented and validated by field investigation. The localization of settlement subdivisions, the second criterion of stratification, was established using administrative information, the SPOT 5 image and field observations. The regular settlements are characterized by a network of hierarchical streets and the presence of basic services and infrastructures as electricity and water. In contrast, the irregular settlements are defined by an important but disorganized network of tracks and the absence of services and infrastructures. At the conclusion of this exercise, urban stratification comprised four types of urban situations: "regular settlements with average building density", "regular settlements with high building density", "irregular settlements with average building density" and "irregular settlements with high building density" [15].

In each of the four principal areas identified, two sub-areas were chosen by convenience sampling as representative of their geographical location in the city (central or peripheral) and age of housing construction (old or recent). At the second level of sampling, a random selection of households was chosen. In regular sub-areas, yards were randomly selected through cadastral maps. In irregular sub-areas, random sampling could not be applied because there was no cadastral map, so households were selected by the "door-to-door" approach used in the standard Expanded Program of Immunisation (EPI) sampling method [16]. In brief, from each randomly selected starting point, the closest household was chosen and checked for compliance with the inclusion criteria. From this first household, the interviewers moved to the next household according to well-defined rules [16] until four households had been randomly chosen. The same process was repeated from other starting points until the required number of participants was obtained.

As the whole research program focused on the impacts of urban life on disparities in health, an "urban criterion", the duration of residence in Ouagadougou of the head of the household, was taken into consideration. In each randomly selected yard, households were eligible if the head of the household had been born in Ouagadougou or had lived in Ouagadougou for more than five years. In addition, the householder had to be over 34 years old to comply with the WHO standard monitoring group for health conditions among adults. In each qualified household, eligible participants were women of any age and men of 35 years or older. All participants were required to have kinship or marital relationships with the head of the household.

### Data collection

Data were collected by face-to-face field interviews, based on structured questionnaires, conducted either in households or in temporary medical examination centres. Interviewers fluent in the local language were given an intensive three-day field-training program. The survey instrument was evaluated for face validity and pilot tested before use.

### Conceptual framework

In 1973, Andersen and Newman [17] proposed a framework for evaluating the utilization of health care. This model assumes that a person's use of health services is a function of predisposing, enabling and need factors. Predisposing characteristics include gender, marital status, educational level, occupation, length of time in the community and health beliefs. Health beliefs – such as attitudes, values and knowledge of the dental care delivery system – are often influenced by cultural values. Enabling resources refer to attributes specific to the individual or the community (e.g. income, social network, access to regular source of care). Need variables reflect illness levels that require the use of services. Needs can be perceived by the individual and are influenced by cultural beliefs and values (e.g. perceived health status, disease severity, limitation of activity) [18]. In 1997, an expanded version of this model was developed as a conceptual framework for the WHO International Collaborative Study of Oral Health Outcomes (ICS II), which was undertaken in selected industrialized countries [4]. This theoretical model was derived by integrating existing oral health behaviour and oral health status models with the general health model of Andersen and Newman. In the present study, we adapted the individual-level determinants proposed in both models to an urban setting of Burkina Faso.

We defined a set of independent individual-level variables that may influence utilization of oral health care: (1) predisposing socio-demographic and health beliefs factors, which can be either modifiable (e.g. education, marital status or health attitudes such as perceived general health status and seriousness of oral disease, importance of oral health, benefits of brushing, or perceived barriers to obtaining oral health care) or non-modifiable (e.g. age or sex); (2) enabling characteristics, which refer to specific attributes of the individual or the community in which the individual lives (e.g. level of income, residence, family size, integration in urban life, social support) and may affect ability to access the health care system; and (3) need factors, which reflect the perceived need for oral health care (patient's perceptions of illness, impairment of quality of life) or the self assessment of health status.

Furthermore, because of the complexity of assessing the socio-economic level of households, a composite index for material living conditions of households was computed using the SPSS 13.0. Module of Multiple Correspondence Analysis. This index was based on ownership of certain household assets such as refrigerator, television, motorcycle and car. Finally, the dependent variables were: whether individuals had experienced an oral health problem during the past twelve months; whether they practised self-medication; and whether they made a dental visit. No distinction was made between preventive and curative visits.

### Statistical analysis

The data were processed and analyzed by SPSS 13.0. First, characteristics of the study sample were described, then frequency distributions were used to highlight the socio-demographic status of participants who had or not experienced an oral problem within the past twelve months. Secondly, chi-square tests were used to detect statistically significant proportions of people who used oral health facilities only, people who practised self-medication only, and people who did not seek treatment at all, in relation to various independent variables. Thirdly, logistic regression was performed in order to estimate the relative risk (Odds Ratio) of the independent variables explaining the use of oral health services within one year. The full regression model was specified on the basis of the theoretical model (the Anderson and Newman model). Our general approach was to test the predisposing characteristics first, then the enabling variables, and then the need variables. Estimates are presented with 95% confidence intervals. Results were weighted using the sampling proportions in order to reflect the population in the strata studied. There were no major differences in estimates when the sample data rather than weighted data were used. In addition, a multi-colinearity diagnostic statistic was derived from the Variance Inflation Factor for each variable found to be significantly associated with our dependent variable.

## Results

### Sampling characteristics

The overall rate of participation of people in the study was 65.0%. The final sample consisted of 3030 adult city-dwellers aged 15 years or over and living in 1816 households of Ouagadougou. For variables such as age, gender, religion, ethnic group and number of years spent in Ouagadougou, we compared non-respondents and respondents. No differences in participation rate were found among socio-demographic groups, except for the relationship with gender. The participation rate was lower among men than women because men left the house early in the morning to go to work and came back late in the evening. Twenty-seven percent of the respondents were born in Ouagadougou and 44.0% had been resident in the city for 5–20 years. About two-thirds (67.4%) of the sample were women.

### Experience of oral health problems

Overall, 27.7% indicated that they had experienced an oral problem within the past 12 months. Women in general, and first spouses of the head of household in particular, seemed to be most affected (p < 0.05) by oral problems (Table 1). Among those declaring oral problems, a high proportion (62.1%) reported the experience of toothache caused by hot, cold or sweet food items while 43.6% of adults indicated pain when they chewed hard items. In addition, 27.3% of respondents stated that they had trouble sleeping because of pain and 21.0% of adults reported having fever and an abscess in the mouth.

### Decision-making in seeking oral health care

**Figure 1:**
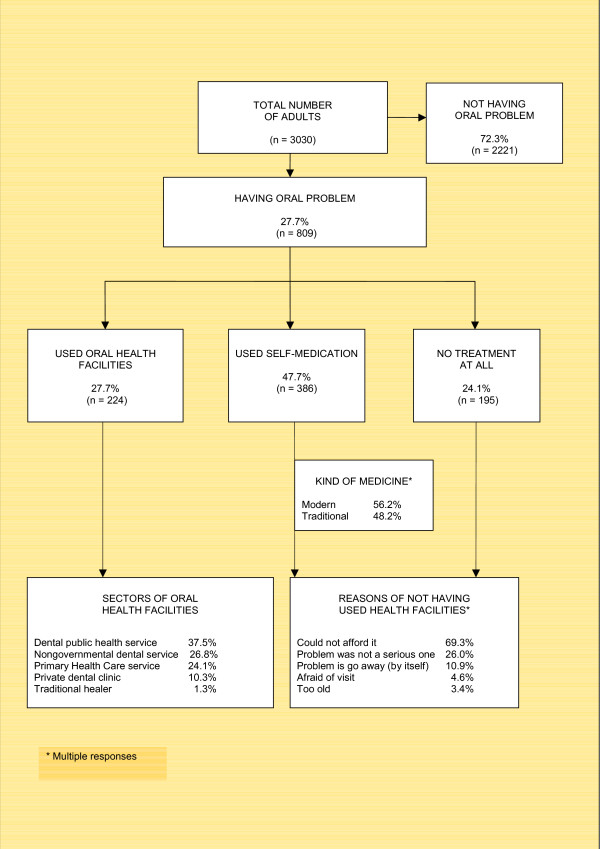
Responses to oral symptoms.

Among individuals who reported having experienced an oral health problem, only 27.7% used oral health facilities, 47.7% reported self-medication and 24.1% chose no treatment at all (Figure 1). In addition, 54.5% of the adults who used oral health facilities claimed that oral problems limited their usual activities, against 38.3% and 24.1%, respectively, of those who used self-medication or no treatment at all. Among adults who consulted medical providers, 64.3% visited public or nongovernmental dental services but 24.1% of them attended a primary health care service with no dental staff. Only 1.3% declared that they had seen a traditional healer. Of the respondents with oral health problems who did not visit an oral health care facility, 69.3% emphasized that it was because of the high cost. Those who relied on self-medication were more likely to report use of modern medicines (56.2%) than traditional ones (48.2%).

### Predisposing characteristics

Table 2 shows the results of bivariate analysis of oral health care choices among adults who reported that they had experienced an oral problem during the previous 12 months in relation to selected predisposing variables. High school graduates, employees, and persons with relatively high material living conditions and residing in a house constructed of modern materials were more likely to visit services for oral health care. No statistically significant differences in illness-related behaviour were found by sex or age. The results also suggest that adults were more likely to use oral health facilities if they perceived oral disease to be important as other health problems or considered fluoride prevents tooth decay and dental visits to have important benefits. Persons who believed that going to the dentist is synonymous with pain were also the most likely to visit oral health providers.

### Enabling and need characteristics

Table 3 outlines the choices of oral health care among adults who reported that they had experienced an oral problem during the previous 12 months in relation to selected enabling and perceived needs variables. Overall, residents of regular areas with high building density and adults with long urban life experience were more likely to use oral health facilities. In addition, participants with active social networks were most likely to have made a visit. Respondents who were very satisfied with the appearance of their teeth and persons who declared that oral problems limited their usual activities were more likely to visit oral health care providers.

### Multivariate analysis

The results of logistic regression analysis of the predictors for using oral health care facilities are summarised in Table 4. Various predisposing factors significantly increased the probability of using oral health care facilities, including Christian affiliation and moderate or high material living conditions. Adults in the "25–34" age group were likely to use oral health facilities. The two indicators of health beliefs were significantly associated with use of oral health services. First, adults who perceived oral disease to be just as important as general diseases were most likely to have made a visit. Second, those who disagreed with the statement that going to the dentist always entails pain were less likely to use oral health facilities. In addition, persons with active social networks and individuals with motorised transport had a higher probability of using oral health care facilities. All other factors being equal, people with the highest perception of impairment in the event of oral problems had a very high probability of having made a visit.

## Discussion

Our data on urban people in Burkina Faso show that oral self-care is practised in accordance with both traditional and modern self-medication cultures. Impairment of function and poor quality of life are also observed in high proportions of individuals who claimed to have their usual activities restricted by oral problems. Moreover, we have identified socio-economic and socio-cultural factors as potentials barriers to the use of oral health facilities. Relatively little is known about oral health service utilization patterns among populations of African countries. The intention of the study was to analyse responses to illness episodes and oral health seeking behaviour among adult city-dwellers in a sub-Saharan country (Ouagadougou, Burkina Faso). In particular, an aim of this investigation was to evaluate the association between utilization of oral health facilities and certain predisposing, enabling and need barriers and facilitators.

In developing countries, random sampling is largely impossible because of the lack of census lists or valid population registers, so alternative procedures are needed to obtain representative samples. In irregular sub-areas, few sampling methods are available for surveying households. In the present study, we applied the "door-to-door" method used in the standard Expanded Program of Immunisation (EPI) developed by WHO [16]. Although this method suffers from a number of disadvantages and was initially developed to measure the coverage of the childhood immunization programmes and nutritional status, it has been widely and successfully used for other purposes in low income countries [19,20]. Furthermore, the response rate is acceptable and only minor differences in socio-demographic profiles among participants and non-participants were noted. Despite the limitations, for those members of the study population who met the eligibility criteria for the study, the data allow reasonable inferences to be drawn about the factors most significantly associated with the use of dental services in the study area.

Studies examining the utilization of oral health care service have measured dependent variables in different ways [3]. The percentage of persons reporting or not reporting a visit to a dentist within the past year is one commonly used parameter for assessing the utilization of dental services. In the context of developing countries where dental visiting habits are primarily orientated towards relief of symptoms or pain rather than preventive care, it seems particularly relevant to express data concerning utilization by people who have experienced oral trouble within the past year.

Compared with western industrialized countries, where about 40–80% of the adults would have visited a dentist within one year [3,4,21], the use of professional oral health care services is alarmingly low in Ouagadougou and self-medication appeared to be an important alternative source of care for the participants in the present study. Despite differences in study design, this pattern accords with previous surveys carried out in Nigeria [8], Ivory Coast [7] and South Africa [6]. It is interesting to note that urban studies conducted in Burkina Faso [22] and in Congo [23] revealed that adults reporting general illness symptoms also had low attendance at professional health services while self care was common practice.

Our study also emphasises the association between religious affiliation and the use of health facilities. This somewhat surprising finding may reflect the historical basis of the implementation of health services in Ouagadougou since the colonial period. Christian missionaries (Protestant or Catholic communities) have played a major role in providing of health care [24]. In Ouagadougou, the current oral health care services are mainly provided by nongovernmental not-for-profit health centres run by missionaries [14]. Similarly, a relationship between religion and the utilization of maternal and child health services was found in a recent study in Ghana, a country that borders Burkina Faso [25]. The authors speculated that religion represents a social structural attribute that may influence patterns of maternal health utilization.

The socio-economic level of the household is a key determinant of health seeking behaviour [3,26-28]. In our study, the socio-economic standard of the household was measured by a proxy index of material living conditions, which was strongly associated with the use of health care services. In addition, the bivariate analysis showed an association between the choice of oral health care and the regular settlements. The population groups living in irregular settlements were less likely to visit health professionals and also more likely not to seek treatment at all. The multivariate analysis did not confirm this pattern because of the inter-correlation between residential area and the index of material living conditions.

The perceived importance of oral health problems emerged as a significant factor in health service utilization in our study. It was not surprising that adults who paid less attention to oral diseases compared to general diseases were unlikely to seek health services. The perceived severity of symptoms was shown to be an essential determinant of treatment choice. This result is consistent with research showing that cultural beliefs and values may act to reinforce or inhibit the use of health services, depending upon the reference group values [4,29]. Fear and anxiety are often suggested as barriers to use of dental services [30]. This was not the case in the present study, as those who were afraid of dentists were more likely to use dental services. This reflects the fact that dental visits are very unpleasant because the service often rendered for pain relief is extraction of teeth. This observation accords with findings about oral health service utilization among adults in China [31,32].

Access to health care facilities seems to be less of a problem in urban than in rural areas, although travel expenditure tends to increase rapidly in a large city like Ouagadougou. Furthermore, it is worth noting that oral health services are limited and mainly established in the central part of the city [14]. This may help to explain why having a moped or other vehicle appeared to be an enabling factor in using oral health care facilities. Thus, although distance in the big cities of developing countries may not represent a major problem for access to general health facilities [22], it is still a potential barrier to the use of oral health services.

The present study also indicates that active participation in social networks is an important determinant of oral health seeking behaviour in the event of oral affliction. The social networks mechanisms consist of advising about or facilitating access to particular health providers. Furthermore, the number of years spent in the city or having been born in Ouagadougou were not associated with use of oral health services. This may suggest that use of oral health services depends more on participation in urban networks than on the duration of urban life experience. Such findings corroborate various oral health care utilization studies carried out in industrialized countries [3,33]. Other studies conducted both in both developed and developing countries and focusing on general health seeking behaviour have also emphasized the influence of social networks as predictors of health care utilization [34,35].

## Conclusion

Our data on the urban adult population in Burkina Faso showed that oral self-care accords with both traditional and modern self-medication practices. Out-of-pocket expenses for health services in general and for oral health services in particular may lead to widespread self-medication among the populations of developing countries. In addition, socio-cultural factors have been identified as significant in relation to use of oral health services. These factors can help identify groups "at risk" and assist in planning public health intervention programs.

For most urban centres in sub-Saharan Africa, the capacity of the oral health system is still insufficient to match the needs of all individuals suffering from pain or discomfort and to serve the population at large, especially in deprived communities. National health authorities should explore critical issues concerning self-medication for the prevention and treatment of oral symptoms before promoting the potential benefits of affordable alternative sources of care. Furthermore, decision-makers must seek to ensure that access to essential oral health care is improved. Such initiatives should pay specific attention to the poorest population groups and thereby also facilitate their access to health services.

## Competing interests

The author(s) declare that they have no competing interests.

## Authors' contributions

BV participated in protocol development and study design, conducted the field work, analysed and interpreted data, drafted and revised the manuscript. PEP took part in protocol development study design, supervision of data analysis, and writing and editing of the manuscript. FF participated in the study design, coordinated the field work and revised the manuscript. PM participated in the statistical analysis and revised the manuscript critically at different stages. JG managed and supervised the data collection and the statistical analysis. SO participated in protocol design and revised the manuscript. MH participated in the study design, in the data collection in the field and revised the manuscript. GS conceived the research program and participated in the writing of the manuscript. All authors contributed to the final version of the manuscript.

## Pre-publication history

The pre-publication history for this paper can be accessed here:



## Figures and Tables

**Table 1:** Socio-demographic characteristics of adults having experienced an oral health problem

Independent variables	n	%
Age group, years (n = 3025)		
15 to 24	267	20.2
25 to 34	689	27.4
35 to 44	1031	27.4
45 to 54	523	27.0
Over 54	515	27.2
Gender (n = 3030)		
Female	2042	27.8^a^
Male	988	24.4
Household marital status (n = 3029)		
Household chief	1088	25.4
First spouse of household chief	1056	29.9^b^
Others members of household	885	24.5
Level of education obtained (n = 3015)		
High	564	24.5
Low	806	27.4
None	1645	27.1
Occupational status (n = 3019)		
Employee	473	24.7
Housewife	743	26.0
Independent workers	1060	28.9
Without occupation	380	26.3
Others	363	25.3
Religion (n = 2982)		
Christian/Animist	1283	26.9
Muslim	1699	26.8
Material living conditions of household (n = 3030)		
Low	1821	28.1
Medium	654	24.8
High	555	24.5
Urban life experience (n = 2948)		
Short (from 5 to 10 years)	582	27.0
Medium (from 11 to 20 years)	718	29.4
Long (more than 21 years)	1648	25.1

^a ^p < 0.05 ^b ^p < 0.01

**Table 2:** Percentages of adults who reported experiencing an oral problem during the past 12 months by choice of oral health care in relation to selected predisposing factors

Independent variables		Used health facilities	Used self-medication	No treatment at all
	n	%	%	%
Level of education obtained				
High	138	41.6^c^	35.7	22.7
Low	221	32.0	49.2	18.8
None	445	21.8	50.9^b^	27.3^a^
Occupational status				
Employee	117	38.5^b^	37.6	23.9
Housewife	193	19.7	53.4	26.9
Independent workers	306	27.5	48.1	23.5
Without occupation	100	27.0	48.0	25.0
Others	92	33.6	46.8	19.6
Religion				
Christian/Animist	345	34.5^c^	40.9	24.6
Muslim	456	23.6	52.7^b^	23.7
Material living conditions of household				
Low	511	19.3	53.4^c^	27.3^a^
Medium	162	35.8	44.4	19.8
High	136	50.5^c^	31.4	18.1
Type of house				
Modern building materials	219	40.6^c^	36.5	21.5
Mixed building materials	434	24.2	50.0	25.8
Traditional building materials (mud)	154	19.5	56.5^c^	23.4
Oral diseases are as important as other health problems				
Agree	671	30.4^b^	47.7	21.9
Disagree	89	14.6	46.1	39.3^b^
Do not know	46	17.4	56.5	26.1
Oral problems cause other general diseases				
Agree	520	29.4	50.6	20.0
Disagree	68	22.1	41.2	36.7^c^
Do not know	207	24.7	44.4	30.9
Fluoride prevents tooth decay				
Agree	258	37.6^c^	40.3	20.9
Disagree	14	35.7	50.0	14.3
Do not know	533	22.9	51.0^a^	25.9
Dental visits to have important benefits				
Agree	596	31.9^c^	46.3	21.8
Disagree	49	23.4	53.2	23.4
Do not know	162	14.2	52.5	33.3^b^
Going to dentist is synonymous with pain				
Agree	353	36.9^c^	41.9	21.2
Disagree	229	33.3	45.8	20.9
Do not know	225	8.5	59.3^c^	32.2^b^

^a ^p < 0.05 ^b ^p < 0.01 ^c ^p < 0.001

**Table 3:** Percentages of adults who reported experiencing an oral problem during the past 12 months by choice of oral health care in relation to selected enabling and needs factors

Independent variables		Used health facilities	Used self-medication	No treatment at all
	n	%	%	%
Urban life experience				
Short (spent from 5 to 10 years)	157	20.6	55.0	24.4
Medium (spent from 11 to 20 years)	211	25.6	46.9	27.5
Long (spent more than 21 years)	414	31.4^a^	45.5	23.1
Residence settlement				
Irregular settlement with HBD*	223	18.8	52.5	28.7^a^
Irregular settlement with ABD**	232	26.7	50.0	23.3
Regular settlement with HBD*	159	42.6^c^	41.3	16.1
Regular settlement with ABD**	195	27.5	45.6	26.9
Social network				
No participation	534	26.2	48.3	25.5
Weak participation	161	24.8	47.9	27.3^a^
Active participation	108	41.7^b^	43.5	14.8
Means of transport in town				
Moped or vehicle (car, taxi or bus)	248	49.4^c^	29.8	20.8
On foot or bicycle	563	22.8	53.7^c^	23.5
Satisfaction with appearance of teeth				
Yes, very satisfied	51	39.2^a^	49.0	11.8
Yes, fair	229	32.4	39.9	27.7
No, dissatisfied	398	24.8	51.9^a^	23.3
No, very dissatisfied	129	25.1	49.2	25.7
Do you have any natural teeth at all?				
Yes	359	24.1	46.6	29.3^b^
No	445	30.9	48.8	20.3
Oral problem caused limitation of usual activities				
Yes	311	39.6^c^	47.8	12.6
No	498	20.5	47.9	31.6^c^

^a ^p < 0.05 ^b ^p < 0.01 ^c ^p < 0.001

* High Building Density

** Average Building Density

**Table 4:** Logistic regression results on the factors associated with using health facilities among adults having experienced an oral problem (n = 809)

Independent variables	Odds ratio	95% CI	*P*-value
*Predisposing socio-demographic factors*			
			
Age group, years			
15–24	1.07	0.44–2.63	*0.877*
25–34	2.65	1.48–4.74	*0.001*
35–44	1.68	0.98–2.89	*0.059*
45–54	1.46	0.78–2.73	*0.242*
Over 54	-		
			
Religion			
Christian/Animist	1.81	1.27–2.56	*0.001*
Muslim	-		
			
Material living conditions of household			
High	3.39	2.11–5.43	*<0.001*
Moderate	2.01	1.29–3.12	*0.002*
Low	-		
			
*Predisposing health belief factors*			
			
Oral diseases are as important as other health problems			
Agree	2.09	1.21–3.62	*0.009*
Disagree/Don't know	-		
			
Going to the dentist is synonymous with pain			
Disagree/Don't know	0.47	0.33–0.66	*<0.001*
Agree	-		
			
*Enabling factors*			
			
Social network			
Active participation	1.84	1.12–3.03	*0.016*
Weak participation	-		
			
Means of transport in town			
Moped or vehicle (car, taxi or bus)	2.15	1.44–3.21	*<0.001*
On foot or bicycle	-		
			
*Perceived need factors*			
			
Oral problem caused limitation or stopped any of usual activities			
Yes	3.43	2.38–4.93	*<0.001*
No/Don't know	-		
